# Phenolic Compounds Enhance Aluminum Tolerance in Chinese Fir (*Cunninghamia lanceolata*) by Regulating Reactive Oxygen Species Homeostasis and Cell Wall Properties Under Aluminum Stress

**DOI:** 10.3390/plants14172658

**Published:** 2025-08-26

**Authors:** Shanshan Xu, Jiahui Wei, Xin Wang, Ruobing Zhang, Jiahua Gao, Xiaoling Li, Chen Wang, Yiquan Ye

**Affiliations:** 1 College of Forestry, Fujian Agriculture and Forestry University, Fuzhou 350002, China; shi_xu_1987@163.com (S.X.); wwmm11233@163.com (J.W.); 13328697017@163.com (X.W.); 18876380637@163.com (R.Z.); 13290769717@163.com (J.G.); lxl220038@163.com (X.L.); 19189377063@163.com (C.W.); 2 State Forestry and Grassland Administration Engineering Research Center of Chinese Fir, Fuzhou 350002, China; 3 University Key Laboratory of Forestry Stress Physiology, Ecology and Molecular Biology of Fujian Province, Fuzhou 350002, China

**Keywords:** aluminum stress, phenolic substances, 2-aminoindan-2-phosphonic acid (AIP), methyl jasmonate (MJ), antioxidant system, cell wall properties

## Abstract

Aluminum (Al) toxicity in acidic soils severely limits the productivity of Chinese fir (*Cunninghamia lanceolata*) plantations. Despite being a crucial timber species in southern China, the regulatory mechanisms underlying phenolic accumulation and Al tolerance pathways under Al stress in Chinese fir remain unidentified. In this study, 5-month-old Chinese fir seedlings were treated with an exogenous phenolic synthesis inhibitor (AIP) and precursor (MJ) to establish the following groups: CK, AIP, MJ, Al, Al+AIP, and Al+MJ. Physiological and biochemical indicator analyses, transcriptome analysis, and protein interaction network predictions were conducted. The findings revealed that phenolic compounds enhance Al tolerance in Chinese fir through two mechanisms: (1) regulation of active oxygen homeostasis (elevating SOD and POD activities, promoting AsA and GSH accumulation, and augmenting total antioxidant capacity); and (2) modulation of cell wall characteristics (increasing pectin content and pectinase activity, and facilitating Al sequestration in the cell wall). Moreover, MJ was found to synergistically enhance these processes, while AIP impeded them. Genes associated with antioxidant enzymes, secondary metabolite synthesis, and cell wall modification were implicated in the regulatory mechanisms. This study provides a theoretical foundation for elucidating the adaptation of Chinese fir to Al toxicity in acidic soil environments, offers insights for enhancing Chinese fir productivity in acidic soils, and presents a novel target for breeding trees with stress resistance.

## 1. Introduction

Chinese fir is an important fast-growing commercial timber species in China, recognized for its rapid growth, high-quality wood, and resistance to pests and diseases, thereby contributing significantly to the country’s forestry production [1]. Long-term forestry practices have shown that aluminum (Al) severely restricts the growth of Chinese fir in acidic soils, resulting in yield declines in replanted stands [2,3]. The intensification of acid deposition and inappropriate fertilization practices have accelerated soil acidification across China [4]. Consequently, the levels of active Al in soils have increased, exacerbating Al toxicity in Chinese fir [5,6]. Therefore, elucidating the adaptive strategies of Chinese fir to Al toxicity is critical for enhancing Al tolerance and mitigating replant-related yield loss.

Al, as the most abundant metallic element and the third most prevalent element in the Earth’s crust, typically exists in stable forms such as aluminosilicate or alumina [7]. However, under acidic soil conditions (pH < 5.5), Al readily dissolves into Al^3+^ ions, which are highly toxic to plants [8,9]. This poses a major constraint to crop productivity in acidic soils, which account for approximately 30% of the world’s arable land and 50% of its potential arable land [5,10]. Al stress disrupts multiple physiological processes in plants, leading to the production of reactive oxygen species (ROS), including superoxide radicals (O_2_^•−^), hydrogen peroxide (H_2_O_2_), hydroxyl radicals (^•^OH), and singlet oxygen (^1^O_2_). These ROS trigger a series of deleterious effects, including (1) membrane lipid peroxidation; (2) altered composition and fluidity of membrane lipids; (3) diminished ion channel activity; and (4) disrupted intracellular ion homeostasis. These effects ultimately cause cellular dysfunction, metabolic imbalance, and impaired plant growth [11,12,13]. To counteract oxidative damage caused by ROS, plants have evolved a series of defense mechanisms comprising enzymatic and non-enzymatic antioxidant systems that evolved through prolonged exposure to stress [14], which help sustain cellular ROS homeostasis and alleviate stress-induced oxidative harm [15]. The enzymatic antioxidant system consists of enzymes, including key enzymes such as catalase (CAT), superoxide dismutase (SOD), and peroxidases (e.g., POD, APX), as well as reductases such as DHAR, GPX, and GR. Additionally, the non-enzymatic antioxidant system comprises low-molecular-weight antioxidants, including ascorbic acid (AsA), glutathione (GSH), and phenolic compounds [6]. These antioxidants can directly scavenge ROS and collaborate with the enzymatic antioxidant system to eliminate ROS, interrupt chain reactions, and reduce Al-induced oxidative damage [16,17].

Phenolic compounds, widely distributed important secondary metabolites in plants, exhibit strong antioxidant activity due to their distinctive molecular structure, which features hydroxyl groups attached to benzene rings, facilitating efficient electron/H^+^ transfer capabilities [18,19]. As a result, phenolic compounds often demonstrate higher antioxidant efficacy than AsA and GSH [20]. Accumulating evidence highlights the significant role of phenolic compounds in scavenging ROS in plants under abiotic stress. For instance, application of naringenin reduces ROS accumulation in the leaves of salt-stressed kidney beans [21]. Similarly, exogenous treatment with chlorogenic acid enhances antioxidant capacity in apple plants and improves the ROS-scavenging ability of leaves, which ultimately mitigates herbicide-induced oxidative damage and reverses photosynthetic inhibition [22]. Notably, recent studies have shown that Al can significantly increase the accumulation of polyphenolic substances in lettuce [23]. This accumulation enhances both enzymatic and non-enzymatic antioxidants, improving the ROS scavenging capacity of the lettuce and reducing oxidative damage caused by Al stress [23]. Collectively, these findings highlight that phenolic-compound-mediated regulation of ROS homeostasis in plants plays a crucial role in enhancing plant tolerance to adverse stress conditions. A previous study demonstrated that Al stress increases total phenol content in roots and leaves of Chinese fir. Transcriptome analysis further identified phenylpropanoid metabolism as the predominant pathway for the accumulation of phenolic compounds in the roots of Chinese fir under Al stress. These observations suggest that phenolic metabolism may be crucial for mediating Al stress tolerance in Chinese fir. However, the regulatory mechanisms underlying Al-induced phenolic accumulation and their physiological impact on Al tolerance in Chinese fir remain largely unknown. Elucidating these mechanisms is, therefore, essential for understanding the adaptive strategies employed by Chinese fir in Al-toxic environments.

The cell wall serves as the primary site for Al accumulation and plays a critical role in regulating Al tolerance [24,25,26,27]. The presence of negatively charged carboxyl and phosphate groups within cell wall polysaccharides promotes Al binding, with pectin, which is rich in carboxyl groups, and is recognized as the principal site for Al binding among cell wall polysaccharides [28,29]. For instance, the Al-sensitive rice genotype Zhefu802 exhibited a notable increase in Al-binding capacity due to elevated pectin methylesterase activity, leading to enhanced pectin de-esterification and an augmented presence of free carboxyl groups [24]. Conversely, the Al-tolerant genotype Nipponbare demonstrated reduced Al binding by maintaining high pectin methylesterification levels [24]. In addition to pectin, hemicellulose has also been recognized as a significant Al binding site. Although hemicellulose is generally considered to have limited Al-binding ability due to its low charge, a study of *Arabidopsis thaliana* showed that it can adsorb up to 45% of the total Al content in the cell wall [25]. Al stress has been found to inhibit xyloglucan endotransglucosidase (XET) activity by suppressing the expression of xyloglucan endotransglucosidase (*XTH*) genes (*AtXTH14*, *AtXHT15*, and *AtXHT31*), thereby altering the Al-binding capacity of the cell wall [27]. Furthermore, another study indicated that, in Chinese fir, the Al-tolerant genotype YX01 maintains a high degree of pectin methylation by reducing pectin and hemicellulose contents, which decreases the number of available Al-binding sites [30]. The above-mentioned results suggest that dynamic modifications of cell wall components are critical for regulating Al-binding capacity and, consequently, plant Al tolerance. Additionally, the metabolism of phenolic compounds may play an important role in regulating Al tolerance in Chinese fir. However, the adaptive regulatory mechanisms, through which phenolic compounds influence cell wall composition under Al stress, remain poorly understood.

This study focused on Chinese fir, employing a combination of physiological measurements and transcriptome analysis under different treatments (CK, AIP, MJ, Al, Al+AIP, and Al+MJ) to systematically investigate the physiological response processes associated with phenolic metabolism regulation mediated by exogenous MJ/AIP. The study also identified enriched pathways, functional annotations, and expression patterns of differentially expressed genes across various comparison groups. These findings provide foundational insights into the phenolic-compound-mediated regulation of Al tolerance in Chinese fir and offer valuable information for understanding the adaptive strategies employed by Chinese fir against Al toxicity.

## 2. Results

### 2.1. Effects of Al, AIP, and MJ on Al Accumulation, Root Growth, and Key Phenolic Metabolism Enzymes

**Figure 1:**
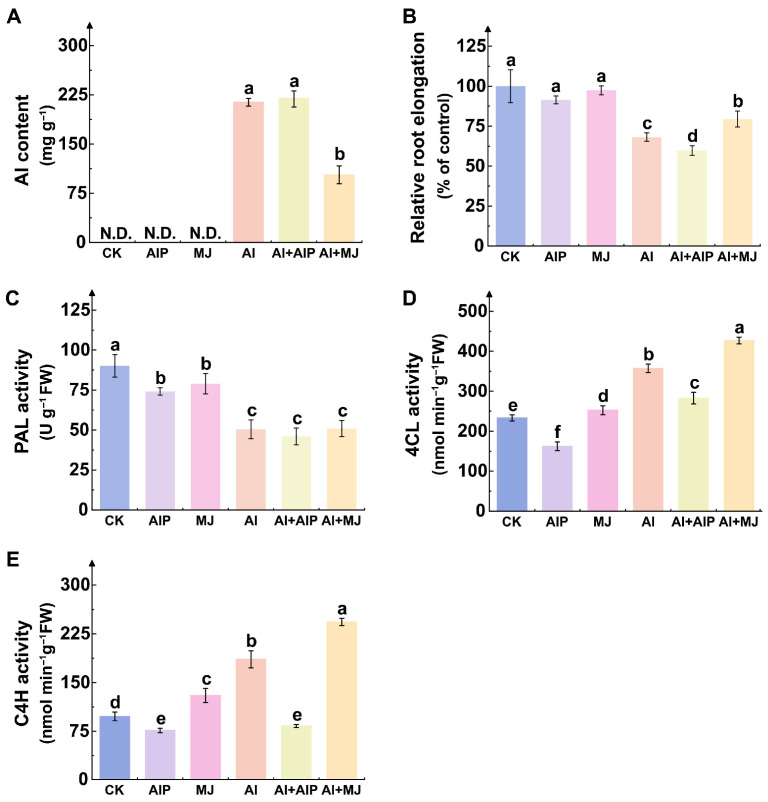
Effects of aluminum (Al), AIP, and MJ on Al content, root growth, and the activities of key enzymes involved in phenolic metabolism in Chinese fir seedlings. Five-month-old Chinese fir seedlings were cultured for 7 days under the following treatments: CK (normal nutrient solution, pH = 4.5), AIP (CK + 0.25 nM AIP, pH = 4.5), MJ (CK + 50 nM MJ, pH = 4.5), Al (CK + 500 μM AlCl_3_, pH = 4.5), Al+AIP (Al + 25 μM AIP, pH = 4.5), and Al+MJ (Al + 50 μM MJ, pH = 4.5). Root tips were harvested to determine Al content (**A**), relative root elongation (**B**), and the activities of PAL (**C**), 4CL (**D**), and C4H (**E**). Statistical analysis was performed using ANOVA, with different lowercase letters above histogram indicating significant differences among treatments (*p* < 0.05). N.D. represents not detected.

To clarify the regulatory role of Al-induced phenolic compound accumulation in Al tolerance of Chinese fir, this study applied AIP and MJ exogenously. Data analysis revealed that, compared with Al treatment alone, Al+AIP treatment increased Al accumulation in the root tips by 2.37%, whereas Al+MJ treatment significantly reduced Al accumulation by 52.60% (*p* < 0.05) (Figure 1A). These results confirm that exogenous MJ effectively inhibits the accumulation of Al ions in the root tips of Chinese fir. Al stress markedly suppressed root growth, and exogenous AIP further intensified this suppression, whereas MJ significantly alleviated Al-induced root inhibition (Figure 1B).

Under different Al treatments, the activity of key enzymes involved in phenolic metabolism in the root tips of Chinese fir showed significant changes (Figure 1C–E). PAL activity responded specifically to Al stress (Figure 1C). Compared with the CK group, PAL activity was reduced to varying degrees under Al, Al+AIP, and Al+MJ treatments (Figure 1C). Specifically, Al treatment resulted in a significant decrease by 44.08% (*p* < 0.05); compared with Al treatment, Al+AIP further inhibited activity by 8.81%, while Al+MJ slightly increased activity by 0.92% (Figure 1C). In contrast, 4CL activity increased across all three treatments (Figure 1D). Al treatment increased 4CL activity by 53.27% compared with CK, while Al+AIP decreased it by 20.82% compared with Al, and Al+MJ increased it by 19.58% (Figure 1D). The activity of C4H exhibited the most pronounced changes. Under Al treatment, C4H activity increased by 89.53% compared with CK (Figure 1E). However, Al+AIP reduced it by 55.15% compared with Al, while Al+MJ increased it by 30.83% (Figure 1E). These results indicate that the activities of key enzymes involved in phenolic metabolism show differentiated responses in Chinese fir under Al stress. Furthermore, AIP and MJ exert significantly different regulatory effects on the activities of these enzymes.

### 2.2. Effects of Al, AIP, and MJ on Protein and Proline Contents

**Figure 2:**
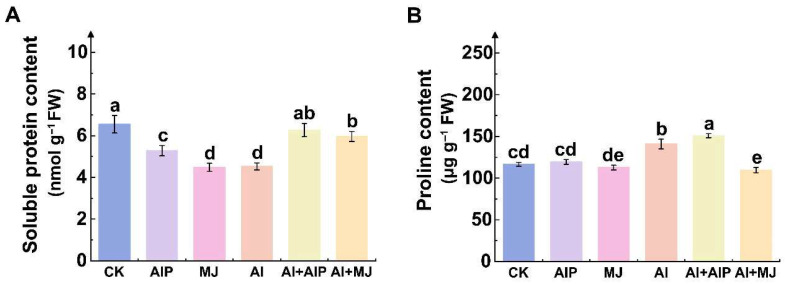
Effects of Al, AIP, and MJ on root protein and proline contents under Al stress. Five-month-old Chinese fir seedlings were cultured for 7 days under the following treatments: CK (normal nutrient solution, pH = 4.5), AIP (CK + 0.25 nM AIP, pH = 4.5), MJ (CK + 50 nM MJ, pH = 4.5), Al (CK + 500 μM AlCl_3_, pH = 4.5), Al+AIP (Al + 25 μM AIP, pH = 4.5), and Al+MJ (Al + 50 μM MJ, pH = 4.5). Root tips were collected for the determination of soluble protein (**A**) and proline (**B**) contents. Statistical analysis was performed using ANOVA, and different letters above histogram indicate significant differences among treatments (*p* < 0.05).

Under Al stress, the soluble protein content in the root tips decreased by 30.89% compared with the CK group (Figure 2A). However, the exogenous application of AIP or MJ significantly reversed this declining trend (*p* < 0.05), increasing the soluble protein content by 38.58% and 31.86%, respectively, compared with the Al treatment (Figure 2A). The results of the free proline determination demonstrated that, compared with CK, proline content increased by 20.90% under Al stress (Figure 2B). In comparison to the Al treatment alone, MJ reduced the proline level by 22.26%, while AIP slightly enhanced the proline content by 2.37% in root tips (Figure 2B).

### 2.3. Effects of Al, AIP, and MJ on Oxidative Damage

**Figure 3:**
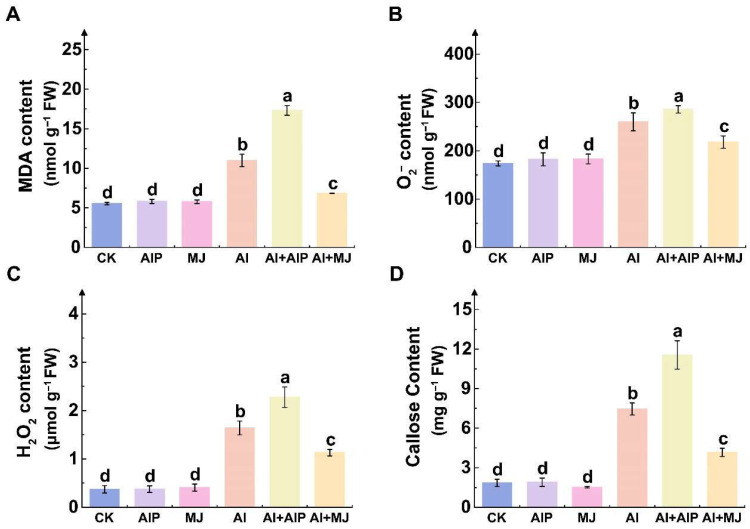
Effects of Al, AIP, and MJ on oxidative damage in Chinese fir seedlings. Five-month-old Chinese fir seedlings were cultured for 7 days under the following treatments: CK (normal nutrient solution, pH = 4.5), AIP (CK + 0.25 nM AIP, pH = 4.5), MJ (CK + 50 nM MJ, pH = 4.5), Al (CK + 500 μM AlCl_3_, pH = 4.5), Al+AIP (Al + 25 μM AIP, pH = 4.5), and Al+MJ (Al + 50 μM MJ, pH = 4.5). After the treatment period, roots were collected, and the contents of malondialdehyde (MDA) (**A**), superoxide anion (O_2_^−^) (**B**), hydrogen peroxide (H_2_O_2_) (**C**), and Callose (**D**) were determined. Statistical analysis was performed using ANOVA, and different letters above histogram indicate significant differences among treatments (*p* < 0.05).

Al stress significantly induced the accumulation of oxidative damage markers (Figure 3). Compared with CK, the levels of MDA, O_2_^−^, H_2_O_2_, and callose increased by 99.22%, 49.38%, 342.31%, and 304.73%, respectively (Figure 3). The Al+AIP treatment further exacerbated oxidative damage, resulting in significant increases in MDA, superoxide anion, H_2_O_2_, and callose content compared with the Al treatment, with increases in 57.68%, 9.93%, 38.84%, and 55.08%, respectively (Figure 3). In contrast, the addition of MJ markedly alleviated the effects of Al stress, reducing the levels of these indicators by 37.63%, 16.14%, 31.08%, and 44.10%, respectively, compared with the Al treatment (Figure 3). These findings suggest that Al stress triggers pronounced oxidative damage and defense responses in the root tips of Chinese fir seedlings. Exogenous AIP exacerbated these stress effects, whereas exogenous MJ effectively mitigated the physiological damage caused by Al toxicity.

### 2.4. Effects of Al, AIP, and MJ on Oxidation Resistance

**Figure 4:**
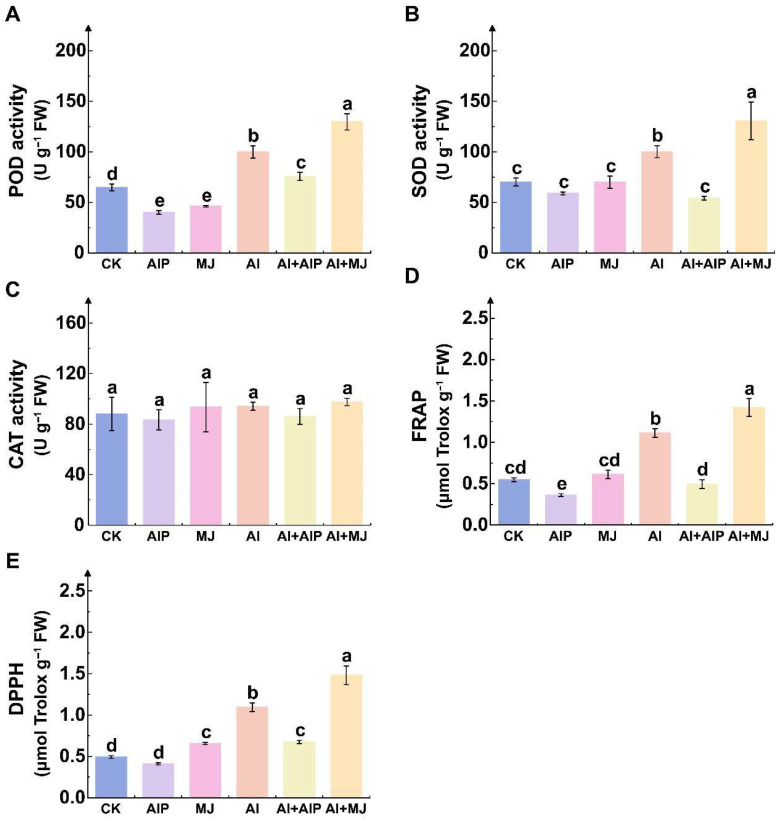
Effects of Al, AIP, and MJ on peroxidase (POD) (**A**), superoxide dismutase (SOD) (**B**), catalase (CAT) (**C**), ferric reducing antioxidant power (FRAP) (**D**), and DPPH (**E**). Five-month-old Chinese fir seedlings were cultured for 7 days under the following treatments: CK (normal nutrient solution, pH = 4.5), AIP (CK + 0.25 nM AIP, pH = 4.5), MJ (CK + 50 nM MJ, pH = 4.5), Al (CK + 500 μM AlCl_3_, pH = 4.5), Al+AIP (Al + 25 μM AIP, pH = 4.5), and Al+MJ (Al + 50 μM MJ, pH = 4.5). After the treatment period, roots were collected, and the above indicators were measured. Statistical analysis was performed using ANOVA, and different letters above histogram indicate significant differences among treatments (*p* < 0.05).

To investigate the effects of AIP and MJ on antioxidant capacity in root tips of Chinese fir seedlings under Al stress, the activities of key antioxidant enzymes were first examined. Al stress significantly induced increased root antioxidant enzyme activities (*p* < 0.05) compared with the CK, with peroxidase (POD), superoxide dismutase (SOD), and catalase (CAT) activities elevated by 54.13%, 42.87%, and 6.98%, respectively (Figure 4A–C). The addition of AIP (Al+AIP) markedly suppressed POD and SOD activities, reducing them by 24.35% and 45.92% relative to Al treatment (Figure 4A,B), while CAT activity showed no significant change, exhibiting only an 8.49% decrease (Figure 4C). In contrast, the application of exogenous MJ (Al+MJ) further enhanced the activities of POD, SOD, and CAT by 29.76%, 30.44%, and 3.70%, respectively, compared with Al treatment alone (Figure 4A–C).

FRAP and DPPH radical scavenging activity were measured to assess total antioxidant capacity. Al stress substantially enhanced root antioxidant activity, with FRAP and DPPH scavenging capacities increasing by 102.94% and 121.58%, respectively, relative to CK (Figure 4D,E). Under Al stress, plants treated with MJ showed higher antioxidant capacity, as indicated by increased FRAP and DPPH values (Figure 4D,E). However, the addition of AIP significantly attenuated the increase in antioxidant activity induced by Al stress, with FRAP and DPPH scavenging abilities reduced by 55.43% and 38.31%, respectively, compared with Al treatment (Figure 4D,E).

### 2.5. Effects of Al, AIP, and MJ on AsA–GSH Cycle

**Figure 5:**
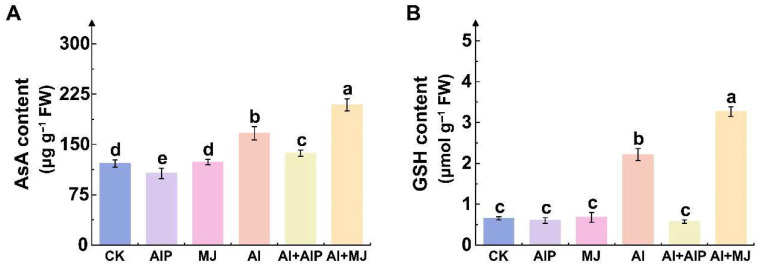
Effects of Al, AIP, and MJ on AsA (**A**) and GSH (**B**). Five-month-old Chinese fir seedlings were cultured for 7 days under the following treatments: CK (normal nutrient solution, pH = 4.5), AIP (CK + 0.25 nM AIP, pH = 4.5), MJ (CK + 50 nM MJ, pH = 4.5), Al (CK + 500 μM AlCl_3_, pH = 4.5), Al+AIP (Al + 25 μM AIP, pH = 4.5), and Al+MJ (Al + 50 μM MJ, pH = 4.5). After the treatment period, roots were collected, and the above indicators were measured. Statistical analysis was performed using ANOVA, and different letters above histogram indicate significant differences among treatments (*p* < 0.05).

Al stress significantly increased the AsA and GSH contents in the root tips of Chinese fir seedlings by 37.09% and 238.59%, respectively (*p* < 0.05) (Figure 5). After adding AIP, the AsA and GSH contents decreased significantly by 17.76% and 74.05%, respectively, compared with Al treatment alone (Figure 5). Conversely, the addition of MJ further increased their accumulation, with AsA and GSH levels rising by 25.64% and 47.51%, respectively (Figure 5), indicating that MJ may synergistically enhance antioxidant synthesis under Al stress.

**Figure 6:**
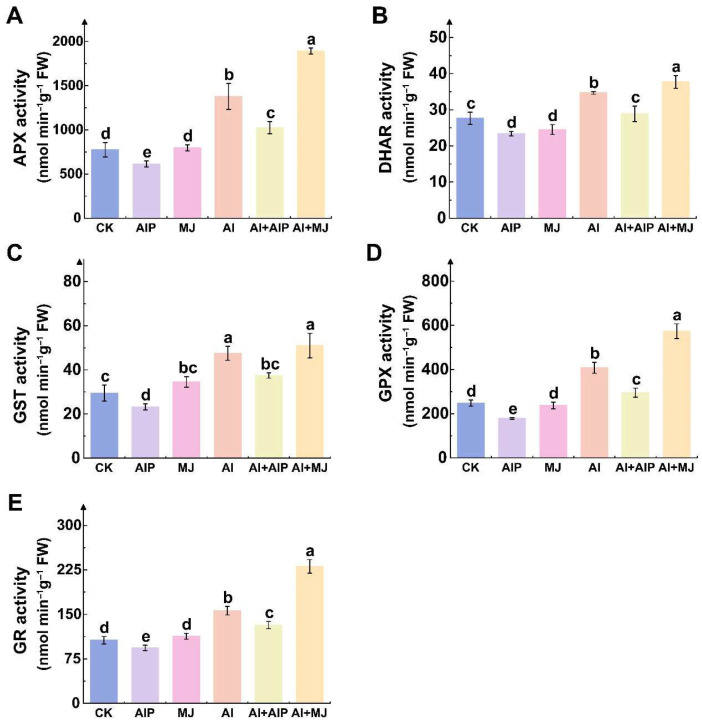
Effects of Al, AIP, and MJ on the activities of key enzymes in AsA–GSH cycle. Five-month-old Chinese fir seedlings were cultured for 7 days under the following treatments: CK (normal nutrient solution, pH = 4.5), AIP (CK + 0.25 nM AIP, pH = 4.5), MJ (CK + 50 nM MJ, pH = 4.5), Al (CK + 500 μM AlCl_3_, pH = 4.5), Al+AIP (Al + 25 μM AIP, pH = 4.5), and Al+MJ (Al + 50 μM MJ, pH = 4.5). After the treatment period, roots were collected, and the activities of ascorbate peroxidase (APX) (**A**), dehydroascorbate reductase (DHAR) (**B**), glutathione S-transferase (GST) (**C**), glutathione peroxidase (GPX) (**D**), and glutathione reductase (GR) (**E**) were measured. Statistical analysis was performed using ANOVA, and different letters above histogram indicate significant differences among treatments (*p* < 0.05).

Analysis of the activities of key enzymes involved in the AsA–GSH cycle revealed that MJ plays a crucial role in regulating the regeneration of AsA and GSH in the root tips of Chinese fir under Al stress. Al stress significantly activated the key enzymes in the cycle pathway, including APX, DHAR, GST, GPX, and GR, which increased by 77.86%, 25.52%, 61.13%, 64.88%, and 46.74%, respectively, compared with the CK (*p* < 0.05) (Figure 6). Compared with Al treatment alone, Al+MJ treatment resulted in varying increases in the activities of APX, DHAR, GST, GPX, and GR by 37.28%, 8.81%, 7.48%, 40.56%, and 47.84%, respectively (Figure 6). Under Al+AIP treatment, the activities of APX, DHAR, GST, GPX, and GR decreased to varying degrees, with reductions of 25.71%, 16.71%, 21.06%, 27.61%, and 15.59%, respectively (Figure 6).

### 2.6. Effects of Al, AIP, and MJ on Cell Wall Fractions

**Figure 7:**
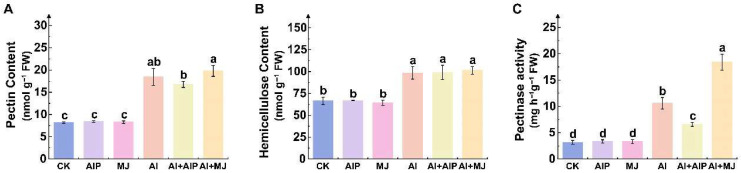
Effects of Al, AIP, and MJ on cell wall components in root tips. Five-month-old Chinese fir seedlings were cultured for 7 days under the following treatments: CK (normal nutrient solution, pH = 4.5), AIP (CK + 0.25 nM AIP, pH = 4.5), MJ (CK + 50 nM MJ, pH = 4.5), Al (CK + 500 μM AlCl_3_, pH = 4.5), Al+AIP (Al + 25 μM AIP, pH = 4.5), and Al+MJ (Al + 50 μM MJ, pH = 4.5). After the treatment period, roots were collected, and the pectin content (**A**), hemicellulose content (**B**), and pectinase activity (**C**) were measured. Statistical analysis was performed using ANOVA, and different letters above histogram indicate significant differences among treatments (*p* < 0.05).

Compared with Al treatment alone, the addition of AIP significantly reduced pectin content in the cell wall by 9.13%, while the addition of MJ increased pectin content by 7.37% (*p* < 0.05) (Figure 7A). Hemicellulose content was not significantly affected by the exogenous addition of AIP or MJ (*p* > 0.05) (Figure 7B). Crucially, pectinase activity decreased by 37.67% in the Al+AIP treatment group compared with the Al group, while it significantly increased by 73.99% in the Al+MJ treatment group (*p* < 0.05) (Figure 7C). These results indicate that the exogenous addition of MJ enhances Al retention in the cell wall by increasing pectin content and pectinase activity, thereby reducing Al transport into the cell and alleviating toxic effects.

### 2.7. Overview of Transcriptome Analysis of Chinese Fir Root Tips Under Different Treatments

Using Illumina high-throughput sequencing technology, this study conducted transcriptome sequencing of RNA samples from the root systems of 12 different treatment groups (CK, Al, Al+AIP, and Al+MJ), with three biological replicates per group. This analysis yielded a total of 94.6 Gb of clean data (Table S1). The GC content of all samples was >43.33%, and the Q30 value was >96.72%, indicating that the data quality met the analysis standards (Table S1). Alignment of the sequencing data to the reference genome revealed effective alignment rates of 86.50–87.20% for CK, 84.82–85.29% for Al, 85.33–85.63% for Al+AIP, and 80.06–80.57% for Al+MJ, confirming the overall effective utilization of the transcriptome data (Table S2). Among the 45,365 expressed genes detected in the 12 Chinese fir root tip samples, the majority of gene expression levels (FPKM values) were distributed within the 0–50 range (Table S3). Pearson correlation coefficients (r^2^) confirmed a strong correlation between biological replicates (r^2^ approaching 1) (Figure S1). Differentially expressed genes (DEGs) were identified using a threshold of Fold Change ≥ 2 and FDR < 0.01. The total number of DEGs for the six comparisons was as follows: CK vs. Al (5546), CK vs. Al+AIP (5165), CK vs. Al+MJ (5191), Al vs. Al+AIP (2078), Al vs. Al+MJ (3477), and Al+MJ vs. Al+AIP (4173) (Figure S2). Among the DEGs in the CK vs. Al comparison, 3099 were upregulated and 2447 were downregulated; in CK vs. Al+AIP, 2758 were upregulated and 2407 were downregulated; in CK vs. Al+MJ, 2260 were upregulated and 2931 were downregulated (Figure S2). In the Al vs. Al+AIP comparison, there were 1001 upregulated and 1077 downregulated DEGs; in the Al vs. Al+MJ comparison, 1090 DEGs were upregulated and 2387 were downregulated; and in the Al+MJ vs. Al+AIP comparison, 2674 DEGs were upregulated and 1499 were downregulated (Figure S2).

### 2.8. GO Functional Annotation of DEGs Between Different Treatments

**Figure 8:**
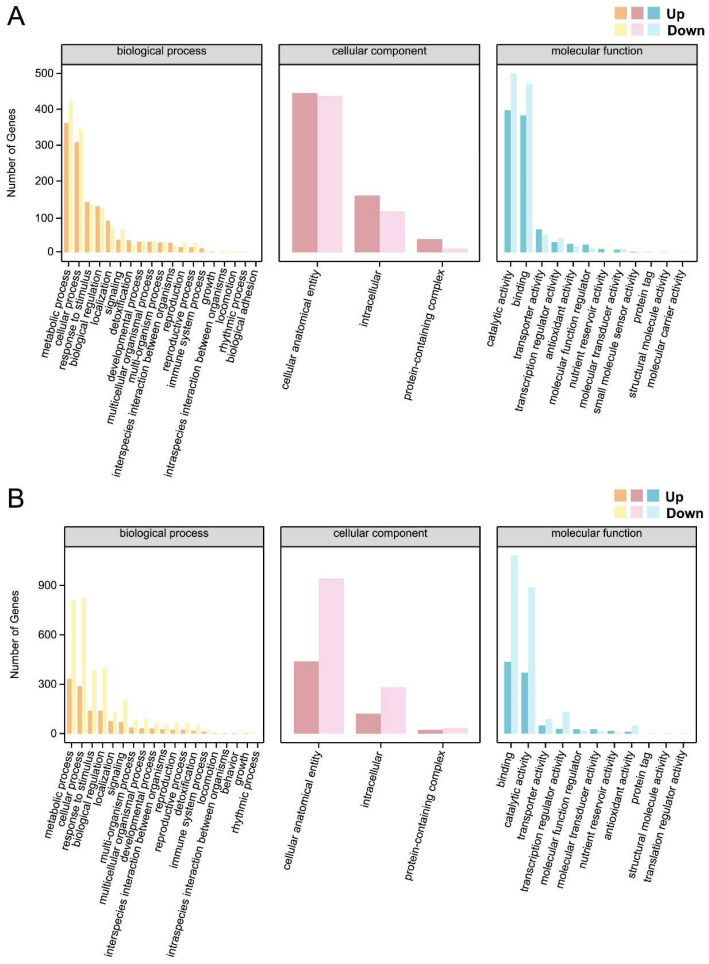
GO annotation of upregulated and downregulated DEGs in the root tips of Chinese fir. (**A**) GO enrichment analysis bar chart of Al vs. Al+AIP. (**B**) GO enrichment analysis bar chart of Al vs. Al+MJ. Yellow, pink, and blue represent biological process, cellular component, and molecular function, respectively. Darker columns represent the number of upregulated DEGs, while lighter columns indicate the number of downregulated DEGs.

In the GO functional annotation, the distribution of DEGs in each comparison group displayed characteristic changes associated with the treatment (Figure 8 and Figure S3). In the biological process category, genes associated with metabolic processes and cellular processes were significantly enriched across all comparison groups, though the patterns of upregulation and downregulation varied. In the CK vs. Al group, 1143 genes were upregulated in metabolic processes, while 993 were upregulated in cellular processes (Figure S3A). In this case, the number of upregulated DEGs exceeded that of downregulated DEGs (Figure S3A). Conversely, in the Al vs. Al+AIP group (metabolic processes: 344; cellular processes: 423) and the Al vs. Al+MJ group (metabolic processes: 809; cellular processes: 823), there were more downregulated than upregulated DEGs (Figure 8A,B). This suggests that Al stress initially triggers widespread metabolic responses, whereas AIP and MJ treatments may alleviate stress by inhibiting these processes.

### 2.9. KEGG Annotation and Enrichment Analysis of DEGs Between Different Treatments

**Figure 9:**
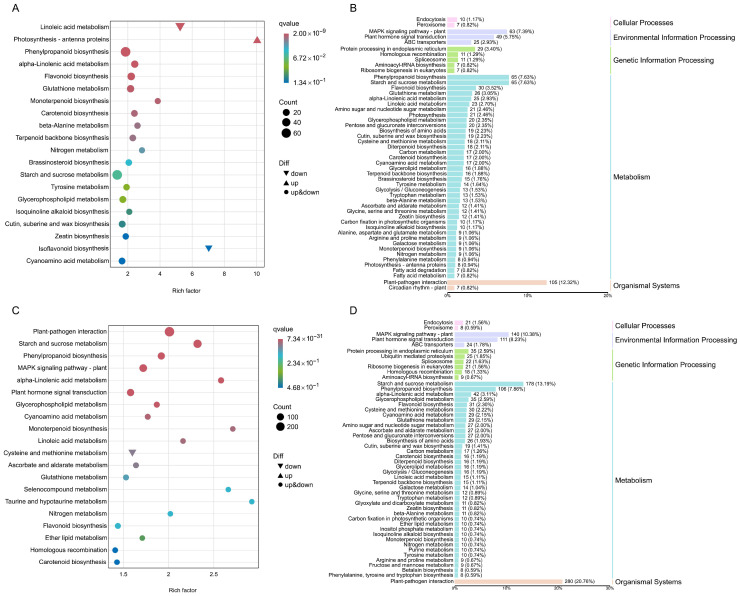
KEGG analysis of DEGs in Chinese fir root tips. KEGG pathway enrichment analysis of DEGs between Al vs. Al+AIP (**A**) and Al vs. Al+MJ (**C**). The x-axis and y-axis represent the enrichment factor and metabolic pathways, respectively. The size of the circles indicates the number of genes enriched in the pathway; larger circles indicate more genes. The smaller the q-value, the redder the color, indicating that the enrichment significance of DEGs in that pathway is more reliable. Triangles represent upregulated DEGs, inverted triangles represent downregulated DEGs, and circles represent both up- and down-regulated DEGs. KEGG classification analysis of DEGs between Al vs. Al+AIP (**B**) and Al vs. Al+MJ (**D**). Different colors represent different pathway types.

KEGG annotation indicated that the DEGs in each comparison group are enriched in five major pathways: cellular processes, environmental information processing, genetic information processing, metabolism, and biological systems. However, their specific distribution characteristics vary depending on the treatment (Figure 9 and Figure S4). The KEGG bubble chart revealed differences in gene expression patterns among the groups within the top 20 significantly enriched (low *p*-value) pathways. In the Al vs. Al+AIP group, the photosynthesis–antenna proteins was the only pathway without downregulated DEGs (Figure 9A). In the Al vs. Al+MJ group, cysteine and methionine metabolism was the only pathway without enriched upregulated DEGs (Figure 9B). In the Al+MJ vs. Al+AIP group, photosynthesis–antenna proteins did not include downregulated DEGs, and biosynthesis of various secondary metabolites-part 2 did not involve upregulated DEGs (Figure S4C). No such expression patterns were detected in the CK vs. Al group (Figure S4A).

In all comparison groups, metabolic pathways were the primary enriched categories of DEGs, among which starch and sucrose metabolism and phenylpropane biosynthesis were core sub-pathways. In the CK vs. Al group, these sub-pathways accounted for 10.13% and 7.05% of the total annotated DEGs, respectively (Figure S4B). In the Al vs. Al+AIP group, sub-pathways constituted 7.63% (Figure 9B). In the Al vs. Al+MJ group, they accounted for 13.19% and 7.86%, respectively, while in the Al+MJ vs. Al+AIP group, they represented 11.32% and 7.23%, respectively (Figure 9D and Figure S4D). Environmental information processing and genetic information processing pathways were the secondary enriched categories, while organismal systems and cellular processes pathways were relatively less enriched. Notably, the organismal systems category had the fewest pathway types; however, the plant–pathogen interaction pathway was the most significantly enriched, accounting for 16.74%, 12.32%, 20.76%, and 18.22% in the CK vs. Al, Al vs. Al+AIP, Al vs. Al+MJ, and Al+MJ vs. Al+AIP groups, respectively (Figure 9B,D and Figure S4B,D). These results suggest that Al stress and MJ/AIP regulation may primarily mediate the Al tolerance response in Chinese fir through pathways associated with metabolism, environmental signal transduction, and pathogen interaction.

### 2.10. Analysis of the Regulatory Mechanisms of DEGs

**Figure 10:**
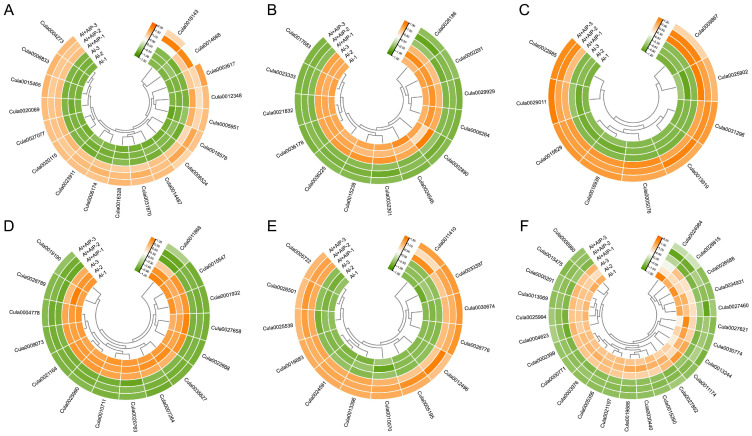
Expression patterns of DEGs in the Chinese fir root tips of Al vs. Al+AIP groups. (**A**,**B**) Upregulated and downregulated expression levels of genes encoding antioxidant enzymes. (**C**,**D**) Upregulated and downregulated expression levels of genes involved in secondary metabolite synthesis. (**E**,**F**) Upregulated and downregulated expression levels of genes involved in cell wall synthesis. All gene expression levels are expressed after normalization adjustment. Clustered by row, the orange gradient indicates upregulated expression levels, and the green gradient represents downregulated expression levels.

**Figure 11:**
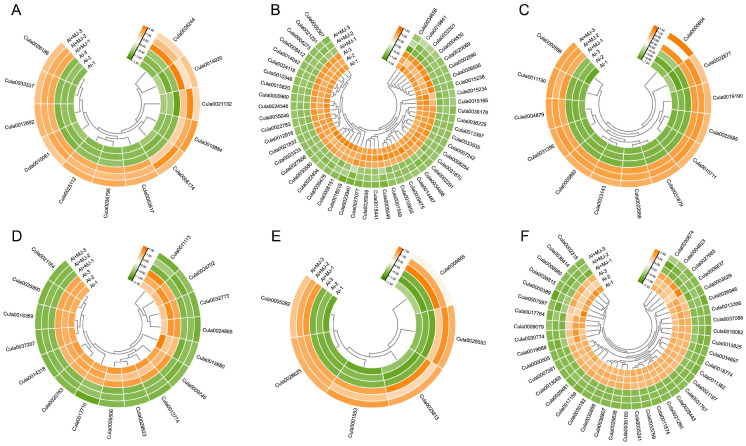
Expression patterns of DEGs in the Chinese fir root tips of Al vs. Al+MJ groups. (**A**,**B**) Upregulated and downregulated expression levels of genes encoding antioxidant enzymes. (**C**,**D**) Upregulated and downregulated expression levels of genes involved in secondary metabolite synthesis. (**E**,**F**) Upregulated and downregulated expression levels of genes involved in cell wall synthesis. All gene expression levels are expressed after normalization adjustment. Clustered by row, the orange gradient indicates elevated expression levels, and the green gradient represents reduced expression levels.

To further investigate the molecular mechanisms underlying the Al tolerance of Chinese fir mediated by phenolic compounds, this study systematically analyzed DEGs in four comparative combinations (CK vs. Al, Al vs. Al+AIP, Al vs. Al+MJ, and Al+MJ vs. Al+AIP) using protein database annotation (Figure 10, Figure 11, Figures S5 and S6 and Table S4). The focus was on elucidating the association between antioxidant enzymes, secondary metabolite synthesis, cell wall modification, and Al tolerance in plants (Table S5).

Analysis of antioxidant enzyme-related DEGs revealed that the CK vs. Al group contained 20 upregulated and 50 downregulated DEGs; the Al vs. Al+AIP group contained 18 upregulated and 13 downregulated DEGs; the Al vs. Al+MJ group contained 12 upregulated and 49 downregulated DEGs; and the Al+MJ vs. Al+AIP group showed a significant activation pattern, with 57 upregulated and 12 downregulated DEGs (Figure 10A,B, Figure 11A,B, Figures S5A,B and S6A,B). These genes are primarily associated with ascorbate oxidase, GST, and POD (Table S4). Among the DEGs associated with secondary metabolite synthesis, the CK vs. Al group contained 41 upregulated and 18 downregulated DEGs, the Al vs. Al+AIP group contained nine upregulated and 15 downregulated DEGs, the Al vs. Al+MJ group revealed 13 upregulated and 16 downregulated DEGs, and the Al+MJ vs. Al+AIP group displayed 27 upregulated and 16 downregulated DEGs (Figure 10C,D, Figure 11C,D, Figures S5C,D and S6C,D). These genes primarily involved flavonoids, including dihydroflavonol 4-reductase (DER), flavonol synthase (FLS), flavone 3′-O-methyltransferase (FOMT), and phenolic modification enzymes, including scopoletin glucosyltransferase (SGT), laccase (LAC), and PAL (Table S4). Additionally, cell wall modification-related DEGs contained 36 upregulated and 39 downregulated genes in the CK vs. Al group; the Al vs. Al+AIP group included 13 upregulated and 24 downregulated DEGs; the Al vs. Al+MJ group had 6 upregulated and 40 downregulated DEGs; and the Al+MJ vs. Al+AIP group had 24 upregulated and 20 downregulated DEGs (Figure 10E,F, Figure 11E,F, Figures S5E,F and S6E,F). These genes primarily cover cellulose synthase catalytic subunit (CESA), cellulose synthase-like protein, pectin methylesterase (PME) and its inhibitor, Endo-1,3-β-D-glucanase, and other key functional genes (Table S4).

### 2.11. PPI Network Analysis of DEGs

**Figure 12:**
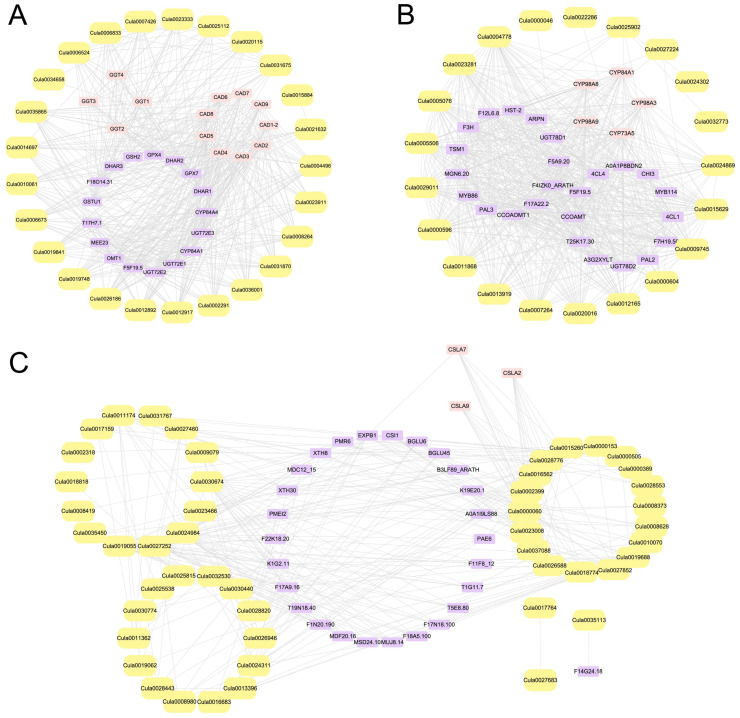
PPI network analysis of DEGs. (**A**) Interaction network of antioxidant enzyme-related DEGs. (**B**) Interaction network of secondary metabolite synthesis-related DEGs. (**C**) Interaction network of DEGs involved in cell wall synthesis. The model plant *Arabidopsis thaliana* was selected as the reference species. The interaction network consists of nodes and edges. Yellow nodes represent the screened DEGs; purple and pink nodes represent interacting proteins retrieved from the STRING database, with pink nodes corresponding to proteins frequently retrieved within the same family. Gray edges represent interactions between proteins.

To analyze the potential synergistic effects of differentially expressed genes (DEGs) encoding antioxidant enzymes, as well as those involved in secondary metabolite synthesis and cell wall synthesis within regulatory networks, a PPI network was constructed based on the STRING database (Figure 12). The PPI networks for the three gene categories were composed of core DEGs and predicted interacting proteins. Among these, antioxidant enzyme-related DEGs exhibit dense interaction relationships with members of the γ-glutamyl transpeptidase (GGT) family and the cinnamyl alcohol dehydrogenase (CAD) family (Figure 12A). Previous studies have confirmed that GGT1 and GGT2 participate in the prevention of oxidative stress by degrading the oxidized form of glutathione disulfide (GSSG) [31,32], while CAD4 and CAD5 are involved in monolignol biosynthesis [33]. These results reveal that proteins interacting with GGT and CAD family members may contribute to these processes through synergistic effects. In the PPI network of DEGs associated with secondary metabolite synthesis, DEGs are densely connected with members of the cytochrome P450 (CYP) family, PAL family, and cinnamic acid-4-hydroxylase (C4H) family, suggesting that their potential cooperative expression may enhance flux through phenylpropanoid and related secondary metabolic pathways (Figure 12B). Notably, all members of the CYP family in the interaction network belong to the A subfamily; among these, the expression of CYP98A9 can influence the metabolism of soluble flavonoids [34]. CYP98A3 catalyzes the synthesis of chlorogenic acid [34]. In Arabidopsis, ATR1/CYP73A5 and ATR2-1/CYP73A5 support the first oxidation step of the phenylpropanoid pathway in a similar manner [35]. In the PPI network of cell wall synthesis-related DEGs (Figure 12C), members of the CSLA family, as the most frequently occurring proteins, showed significant interactions with core DEGs. The CSLA family belongs to a subfamily of the cellulose synthase (CESA) superfamily, with CSLA2, CSLA7, and CSLA9 participating in mannan synthesis, suggesting that this interaction module may influence cell wall structural characteristics by regulating cell wall hemicellulose synthesis [36].

## 3. Discussion

Al stress disrupts cellular metabolic balance by interfering with mitochondrial function, thereby triggering excessive accumulation of reactive oxygen species (ROS) that initiate membrane lipid peroxidation and oxidative damage, ultimately leading to cell death [37,38]. In the present study, the activities of SOD, POD, and CAT in Chinese fir root tips increased markedly under Al stress, yet oxidative damage markers such as H_2_O_2_ and MDA still accumulated (Figure 3 and Figure 4A–C), indicating that the rate of Al-induced ROS production exceeded the scavenging capacity of the antioxidant system. To counteract the ROS burst, plants have evolved a coordinated enzymatic and non-enzymatic antioxidant defense system [14,15]. Exogenous MJ further elevated the activities of SOD, POD, and CAT under Al stress and significantly reduced H_2_O_2_ and MDA contents, demonstrating that MJ-promoted phenolic accumulation enhances the antioxidant capacity of Chinese fir and mitigates oxidative damage (Figure 3 and Figure 4A–C). These findings are consistent with those of Giannakoula et al., who reported that Al-tolerant maize genotypes effectively suppress lipid peroxidation by maintaining higher SOD and POD activities, whereas sensitive genotypes exhibit diminished antioxidant enzyme activity and aggravated oxidative injury [15]. Similarly, Darkó et al. and Yu et al. demonstrated that Al-tolerant wheat genotypes enhance overall antioxidant capacity by upregulating APX and CAT activities under Al stress [39,40].

Beyond direct modulation of the enzymatic antioxidant system, phenolic compounds function as pivotal non-enzymatic antioxidants and signaling molecules that are indispensable for plant stress resilience. Recent studies reveal that phenolics not only alleviate oxidative stress by directly scavenging ROS but also transcriptionally regulate genes, thereby enhancing plant tolerance. For instance, Ali et al. reported that MJ treatment promotes the accumulation of total phenolics and flavonoids in ginseng roots, consequently strengthening antioxidant capacity [41]. In the present study, PAL activity declined, whereas 4CL and C4H activities increased in Chinese fir root tips under Al stress (Figure 1C–E), indicating branch-specific regulation within the phenylpropanoid pathway. We postulate that Al suppresses PAL phosphorylation or selectively activates the downstream lignin-biosynthetic branch, thereby redirecting carbon flux toward 4CL/C4H-dependent monolignol formation [42,43,44]. This contrasts with reports indicating that Al treatment under low phosphorus conditions induces upregulation of PAL expression in young tea leaves [45], and also differs from the response pattern of MJ-enhancing PAL activity in *Brassica napus* under arsenic (As) stress [46], implying that the Al-tolerance mechanism in Chinese fir may exhibit species- and tissue-specific characteristics. In Arabidopsis, MJ is rapidly converted to its active form upon entry into the cell, triggering the COI1–JAZ complex to degrade JAZ repressors; the released MYC2 subsequently activates the transcription factors MYB58/63, which directly upregulate 4CL1/3 and C4H expression to drive monolignol biosynthesis [47]. Notably, we observed that DEGs associated with secondary metabolite synthesis interacted with MYB114 and MYB86, and that 4CL and C4H activities increase under MJ treatment (Figure 1D,E and Figure 12B). Hence, we hypothesize that in Chinese fir, MJ engages an analogous signaling pathway in which ligand binding activates downstream MYB transcription factors, which, in turn, bind to the promoters of 4CL and C4H to upregulate their expression. Furthermore, secondary-metabolite-biosynthesis-related DEGs interact with 4CL1 and 4CL4, further supporting the possibility that 4CL proteins may accelerate the synthesis of phenolic compounds, thereby strengthening the antioxidant system (Figure 12B). However, the specific catalytic mechanisms of 4CL family members for different phenolic substrates, as well as how MJ regulates the expression of 4CL and C4H through upstream signals, remain to be further explored.

The AsA–GSH cycle is a critical non-enzymatic antioxidant system in which AsA and GSH protect plants from Al-induced oxidative damage [48]. We observed that Al stress promotes AsA and GSH accumulation in Chinese fir roots (Figure 5), which enhanced the activities of key AsA–GSH cycle enzymes (APX, DHAR, GST, GPX, and GR) (Figure 6). Conversely, MJ-induced phenolic accumulation further elevates the activities of key enzymes in the AsA–GSH cycle and the contents of AsA and GSH (Figure 5 and Figure 6). Based on the interactions between antioxidant enzyme DEGs and the GGT family, we can infer that glutathione metabolism involving GGT and the activation of key enzymes in the AsA–GSH cycle may have a synergistic effect (Figure 12A). Additionally, genes involved in glutathione metabolism (e.g., *Cula0018441*, *Cula0035336*, and *Cula0003617*) exhibited upregulated expression under Al stress, and genes encoding GSTs (e.g., *Cula0031820* and *Cula0003205*) displayed a similar upregulation pattern (Table S4). This demonstrates that, under Al stress, Chinese fir seedlings likely counteract the imposed stress and enhance intrinsic tolerance by upregulating genes encoding GST and those participating in glutathione metabolism, thereby augmenting the activities of key enzymes in the AsA–GSH cycle and accumulating non-enzymatic antioxidant AsA and GSH. This is consistent with previous research findings, which showed that high levels of AsA and GSH confer tolerance to Al stress in wheat and mitigate ROS-induced oxidative damage [49]. However, the addition of AIP significantly reversed this protective response, whereas the application of MJ enhanced it (Figure 5 and Figure 6). At the genetic level, we observed that *Cula0023333* was downregulated only in the Al vs. Al+AIP and Al vs. Al+MJ comparison groups. We propose that its expression is subject to feedback downregulation in the presence of potent phenolic antioxidants, indicative of a metabolic shift from enzymatic to non-enzymatic defense systems, reflecting the principles of energy conservation and metabolic optimization in plants.

Roots are the primary site of Al stress. Absorbed Al^3+^ not only inhibits root elongation and interferes with the transport of other cations, but also alters cell wall structure and disrupts cellular homeostasis [50,51,52]. The cell wall, which is the main site of Al accumulation, is composed of cellulose, hemicellulose, pectin, and glycoproteins. In the current study, it was observed that, under Al stress, the contents of pectin and hemicellulose increased, and pectinase activity also increased (Figure 7). AIP treatment reduced both pectin content and pectinase activity, whereas MJ treatment markedly enhanced pectinase activity, an effect potentially linked to phenolic-induced expression of cell-wall-remodeling genes (Figure 7). Under MJ treatment, phenolics elicit a broad downregulation of glycosyl hydrolase family members (e.g., *Cula0019062*, *Cula0017159*, and *Cula0026946*) (Table S4), thereby attenuating their roles in degrading cellulose and hemicellulose within the plant cell wall [53]. Zhu et al. supported this finding by observing that Al ions inhibit the expression of *XTH31* genes associated with hemicellulose synthesis, inducing a decrease in hemicellulose content in the cell wall, which weakens Al-binding capacity, reduces the accumulation of Al ions in root cell walls, and enhances Al ion tolerance [54]. The negatively charged functional groups (-COOH, -OH, -SH, etc.) present in cell wall polysaccharides, such as hemicellulose and pectin, can effectively bind with metal ions, immobilizing them in the cell wall and preventing their entry into the cytoplasm, thereby avoiding toxicity. Multiple studies have confirmed that root pectin content is positively correlated with the ability of cell walls to bind metal ions [55,56,57,58]. Furthermore, the ability of cell walls to adsorb metal ions decreases following pectin removal [59]. Thus, we conjecture that, under Al+MJ stress, phenolics enhance pectin–methylesterase activity and preserve hemicellulose, pectin, and other polysaccharides by downregulating glycosyl hydrolase family members; the negatively charged pectin, via its carboxyl groups, chelates Al^3+^ within the cell wall, thereby preventing Al-induced toxicity. Additionally, the interaction between the CSLA family and cell wall modification-related DEGs, along with KEGG enrichment results, implies that these genes are involved in regulating changes in cell wall components (Figure 9D and Figure 12C). Hence, under MJ treatment, the mannan synthesis and pectin metabolism pathways were significantly activated, further enhancing the cell wall’s ability to retain Al and thereby alleviating Al toxicity.

## 4. Materials and Methods

### 4.1. Plant Materials and Treatment

Seeds of Chinese fir (genotype YX06) were obtained from a third-generation seed orchard at Youxi State Forest Farm, Fujian Province, China. The seeds were surface-sterilized with 0.3% (*w*/*v*) KMnO_4_ for 30 min and rinsed thoroughly with deionized water. Buoyant seeds were discarded. The remaining seeds were soaked in warm water (initial temperature of 45 °C) for 24 h. Sterilized seeds were germinated for 28 d on filter paper moistened with 3 mM CaCl_2_. The germinated seedlings were then transferred to a nutrient solution (pH = 4.5) containing 750 μM KNO_3_, 500 μM NH_4_Cl, 250 μM NaH_2_PO_4_, 500 μM CaCl_2_, 125 μM MgSO_4_, 5 μM H_3_BO_3_, 0.25 μM MnSO_4_, 0.25 μM ZnSO_4_, 0.05 μM CuSO_4_, 0.05 μM (NH_4_)_6_Mo_7_O_24_, and 12.5 μM Fe-EDTA for 5 months in hydroponics. The growth chamber environment was maintained at 14 h of light at 25 °C, 10 h of dark at 22 °C, a light intensity of 110 μmol m^−2^ s^−1^, and 75% relative humidity.

Uniform 5-month-old hydroponic seedlings were acclimated in standard nutrient solution (pH = 4.5) for 24 h prior to experimental treatment. To assess the roles of phenolic precursors and biosynthesis inhibitors under Al stress, seedlings were subjected to six treatments for 7 days: CK (Standard nutrient solution, pH = 4.5), AIP (CK + 0.25 nM AIP, pH = 4.5), MJ (CK + 50 nM MJ, pH = 4.5), Al (CK + 500 μM AlCl3, pH = 4.5), Al+AIP (Al + 25 μM AIP, pH = 4.5), and Al+MJ (Al + 50 μM MJ, pH = 4.5). Each treatment included three biological replicates.

### 4.2. Determination of Al Content and Relative Root Elongation

The root systems of Chinese fir were oven-dried at 105 °C for 30 min to deactivate enzymes, followed by drying at 75 °C to reach a constant weight. The dried samples were ground to pass through a 100-mesh sieve. Samples were digested using a microwave-assisted digestion system (ETHOS UP) with a HNO_3_-H_2_O_2_ mixture. The resulting digests were diluted to a known final volume, and root Al concentration was quantified using inductively coupled plasma optical emission spectrometry (ICP-OES; Optima 8000, PerkinElmer Inc., Waltham, MA, USA). Elongation of Chinese fir roots was determined 24 h after treatment initiation, with root length measured before and after treatment. Relative root elongation was calculated as the percentage elongation under each treatment relative to that of the Al-free control [60].

### 4.3. Assay for Key Enzymes Involved in Phenolic Metabolism in Roots

Roots (0.1 g fresh weight) were homogenized in 1 mL of ice-cold extraction buffer (0.1 M Tris–HCl, pH 8.7, containing 1 mM EDTA, 2 mM β-mercaptoethanol, 1% PVP, 15 mM ascorbic acid, and 10% glycerol). The homogenate was centrifuged at 4 °C (12,000× *g*, 30 min) to obtain the supernatant (crude enzyme extract). Subsequently, phenylalanine ammonium lyase (PAL), 4-coumarate: coenzyme A ligase (4CL), and cinnamate-4-hydroxylase (C4H) activities were determined using the PAL, 4CL, and C4H kits (Suzhou Ming Bio-Tech Co., Ltd., Suzhou, China), respectively.

### 4.4. Determination of Oxidative Damage

Proline content was determined using a previously described method [61]. Briefly, the roots were collected and extracted in 3% sulfosalicylic acid and then centrifuged. Subsequently, 1.5 mL of the supernatant was incubated with 2 mL of ninhydrin reagent. The reaction mixture was stirred at 100 °C for 40 min (2.5% (*w*/*v*) ninhydrin, 60% (*v*/*v*) glacial acetic acid, and 40% (*v*/*v*) 6 mol/L phosphoric acid) and then cooled immediately. Absorbance was measured at 520 nm. Proline concentration was calculated from a standard curve.

Malondialdehyde (MDA) content was determined using the thiobarbituric acid (TBA) method [62]. Briefly, 2 g of frozen and ground Chinese fir roots were homogenized in 10 mL of 10% (*w*/*v*) trichloroacetic acid (TCA). The homogenate was centrifuged at 4000 r/min for 10 min at 4 °C, and 2 mL of the supernatant was collected (2 mL of distilled water was used for the blank control). To each tube, 2 mL of 0.6% (*w*/*v*) thiobarbituric acid solution was added, and the mixture was shaken thoroughly. The reaction was carried out in a boiling water bath for 15 min, followed by rapid cooling and centrifugation at 4000 r/min for 15 min. The absorbance of the supernatant was measured at 532, 600, and 450 nm using a spectrophotometer.

The superoxide anion content was determined according to a previous method [63]. In brief, 0.1 g of Chinese fir root samples was placed in a pre-cooled mortar and ground with 1.5 mL of 65 mM phosphate buffer (pH 7.8). The solution was transferred to a centrifuge tube and centrifuged at 5000× *g* for 10 min at 4 °C. Then, 1 mL of the supernatant was taken, and 0.9 mL of PBS buffer and 0.1 mL of 10 mM hydroxylamine hydrochloride were added. After mixing, the mixture was incubated in a 25 °C water bath for 20 min. Following this, 1 mL of the reaction solution was taken, and 1 mL of 1% (*w*/*v*) sulfanilamide solution and 1 mL of 0.02% (*w*/*v*) α-naphthylamine solution were added. The mixture was incubated in a 25 °C water bath for 30 min, and absorbance was measured at 540 nm.

The hydrogen peroxide content was determined according to the method described by RAI et al. [64]: 0.2 g of frozen samples was placed in a pre-cooled mortar and thoroughly ground with 5.0 mL of phosphate buffer (50 mM, pH 6.5). Subsequently, 3.0 mL of the homogenate was mixed with 1.0 mL of 20% (*v*/*v*) sulfuric acid solution containing 0.1% (*w*/*v*) titanium sulfate and centrifuged at 16,000× *g* for 15 min at 4 °C. The absorbance of the supernatant was measured at 410 nm, and the hydrogen peroxide concentration was calculated using an extinction coefficient of 0.28 μmol^−1^ cm^−1^, with the results expressed as μmol g^−1^.

For callose content determination, 0.1 g of Chinese fir root samples were immersed in a 98% (*v*/*v*) ethanol solution and left standing overnight at room temperature. After discarding the supernatant ethanol, 1 mL of 1 M NaOH solution was added to the precipitate, which was ground and transferred to a centrifuge tube, and then heated in a water bath at 85 °C for 15 min. After cooling to room temperature, the mixture was centrifuged at 10,000× *g* for 10 min. Next, 142 μL of the supernatant was taken, and 284 μL of 0.1% (*w*/*v*) aniline blue aqueous solution, 150 μL of 1 M HCl solution, and 420 μL of 1 M glycine-sodium hydroxide buffer (pH 9.5) were added sequentially. After mixing, the mixture was incubated in a water bath at 50 °C for 20 min. Upon completion of the reaction, it was immediately cooled to room temperature in an ice-water bath, and the fluorescence intensity was measured at an excitation wavelength of 400 nm and an emission wavelength of 510 nm using a microplate reader (SpectraMax i3×X, Molecular Devices, San José, CA, USA) [65]. The soluble protein content was determined using the Coomassie Brilliant Blue G-250 staining method, and the absorbance was measured at 595 nm [66].

### 4.5. Antioxidant Enzyme Activity

Catalase (CAT; EC 1.11.1.6) activity was assayed by monitoring the decomposition rate of H_2_O_2_. Peroxidase (POD) activity was analyzed by measuring the oxidation of guaiacol after adding H_2_O_2_. Superoxide dismutase (SOD) activity was determined using the nitroblue tetrazolium (NBT) method [49].

### 4.6. Assessment of Antioxidant Activity in the Root

DPPH activity was determined according to the method described by Kang et al. [67]. The ferric reducing antioxidant power (FRAP) assay was performed following the procedure described by Thaipong et al. [68], which involved mixing 200 μL of root extract with 3 mL of freshly prepared FRAP working solution, reacting in the dark for 30 min, and then conducting the detection.

### 4.7. AsA–GSH Cycle

Ascorbic acid (AsA) content was determined according to the method described by Mukherjee and Choudhurri [69]. Briefly, 0.2 g of fresh roots were ground in an ice bath with 3 mL of 5% (*w*/*v*) metaphosphoric acid. The homogenate was centrifuged at 12,000× *g* for 10 min, and the supernatant was made up to 3 mL with metaphosphoric acid. For measurement, 0.2 mL of the sample solution was mixed with 0.4 mL of 75 mM NaH_2_PO_4_ solution (pH 7.4), followed by the addition of 0.4 mL of 10% (*w*/*v*) metaphosphoric acid, 0.4 mL of 44% (*v*/*v*) phosphoric acid, 0.4 mL of 4% (*w*/*v*) 2,2-dipyridyl (dissolved in 70% ethanol), and 0.2 mL of 3% (*w*/*v*) FeCl_3_. The mixture was shaken and incubated at 37 °C for 1 h, and the absorbance was measured at 525 nm. Reduced glutathione (GSH) samples were extracted using the same method as AsA, and the content was determined using colorimetry at 412 nm using a Hitachi F4600 fluorescence spectrophotometer (Hitachi High-Technologies Corporation, Tokyo, Japan) [70].

To evaluate the activities of key enzymes in the ascorbate–glutathione cycle, fresh root samples were selected, and enzyme extracts were prepared according to the method of Grace and Logan [71]. Briefly, 0.5 g of each sample was ground into a fine powder in a mortar with liquid nitrogen and homogenized in 6 mL of 50 mM KH_2_PO_4_ buffer (pH 7.5) containing 0.1 mM EDTA, 0.3% (*v*/*v*) Triton X-100, and 1% (*w*/*v*) PVP. Ascorbate peroxidase (APX) and dehydroascorbate reductase (DHAR) activities were determined following the method described by Nakano [72]. Glutathione peroxidase (GPX) activity was assayed according to the procedure described by Halliwell [73]. Glutathione S-transferase (GST) activity was measured using the method described by Habig [74]. Glutathione reductase (GR) activity was determined as previously reported [71].

### 4.8. Root Tip Cell Wall Components

Hemicellulose was isolated via hydrolysis using 2 M hydrochloric acid, and its content was calculated based on the concentrations of galactose, mannose, xylose, and arabinose [75]. Pectin content was determined by first extracting pectin using a previously described method, followed by spectrophotometric measurement at 530 nm [76]. For pectinase activity assays, 1 g of root samples was homogenized in a pre-chilled mortar with an equal volume of extraction buffer (0.1 M Tris–HCl (pH 7.5) and 0.5 M NaCl). The homogenate was centrifuged at 12,000× *g* for 10 min at 4 °C, and the resulting supernatant was used for subsequent enzyme activity measurements, which were performed spectrophotometrically at 620 nm [77].

### 4.9. Transcriptome Analysis

After 7 days of treatment, Chinese fir root tip samples were collected, with 3 biological replicates for each treatment. The samples were quickly frozen in liquid nitrogen and stored at −80 °C for subsequent transcriptome analysis. RNA sequencing was commissioned to BioMarker Technologies (Beijing, China) Co., Ltd. The procedures included RNA extraction, followed by detection of purity, concentration, and integrity. After passing the quality controls, the mRNA was fragmented, and cDNA libraries were constructed based on SBS technology using the fragmented mRNA as templates. Finally, transcriptome sequencing was performed on the Illumina NovaSeq6000 high-throughput sequencing platform. The Chinese fir reference genome used in this study was provided by the Chinese Fir Engineering Technology Research Center of National Forestry and Grassland Administration (Cunninghamia_lanceolata.customerv3.0.genome.fa).

### 4.10. Statistical Analysis

Data were initially collated using Excel 2021, followed by multiple comparison analysis with SPSS 27. Analysis of variance (ANOVA; *p* < 0.05) was performed on the corresponding data of each group to determine significance. After generating the sequencing data, bioinformatics analyses were conducted using BMKCloud (www.biocloud.net; accessed on 23 July 2025), including GO analysis, KEGG analysis, and differential gene expression analysis. Relevant graphs and tables were generated using Origin 2024 and TBtools v2.118 [78].

Protein–protein interaction (PPI) network analysis was performed using the STRING website (https://cn.string-db.org/; accessed on 30 July 2025) to predict the interacting proteins of differentially expressed genes (DEGs), with *Arabidopsis thaliana* as the reference species [79]. The parameters were set to a moderate confidence level (0.400) for the minimum required interaction score, and the maximum number of interaction proteins displayed was set to no more than 30 [79]. The TSV result file, in string_interactions_short format, was downloaded, and the PPI network was organized and visualized using Cytoscape software (v 3.10.0) [79].

## 5. Conclusions

This study demonstrated that Al stress significantly induced Al accumulation in the root tips of Chinese fir seedlings, causing oxidative damage, as evidenced by increased levels of MDA, superoxide anions, and H_2_O_2_, and decreased soluble protein content, and activation of defense responses, leading to increased accumulation of proline and callose. Al stress differentially affected the activity of key enzymes in phenolic metabolism, with PAL activity decreasing, and 4CL and C4H activities increasing. AIP significantly inhibited 4CL and C4H activities, while MJ promoted their activities. Al stress activated the antioxidant system and the AsA–GSH cycle, but AIP weakened these systems, while MJ further enhanced them. Under Al stress, MJ promoted Al retention in the cell wall, reducing intracellular Al accumulation; AIP, however, exacerbated Al toxicity. Transcriptome analysis clearly indicated that phenolic compounds enhance the expression of antioxidant enzyme-related genes, such as ascorbate oxidase, APX, and GST, and influence the expression of genes associated with secondary metabolite synthesis and cell wall modification. Protein interaction networks further revealed the interactions between these category-related genes and other proteins. This study revealed that Chinese fir enhances its Al tolerance through a dual mechanism mediated by phenolic compounds under Al stress: (1) regulating ROS homeostasis to alleviate oxidative damage; and (2) regulating cell wall characteristics to reduce intracellular Al accumulation. Phenolic compounds are key regulatory factors in the Al tolerance of Chinese fir. Exogenous MJ can enhance this mechanism, while AIP exacerbates Al toxicity. The pivotal role of phenolic compounds in conferring Al tolerance to Chinese fir highlights potential strategies for improving its adaptation to acidic soils.

## Data Availability

Data are contained within the article or Supplementary Material.

## Supplementary Materials

The following supporting information can be downloaded at the following address: https://www.mdpi.com/article/10.3390/plants14172658/s1, Figure S1: Heatmap of correlation between *C. lanceolata* root tips; Figure S2: Number of upregulated and downregulated DEGs in different control groups of *C. lanceolata* seedling root tips; Figure S3: GO annotation of upregulated and downregulated DEGs in the root tips of Chinese fir; Figure S4: KEGG analysis of DEGs in Chinese fir root tips; Figure S5: Expression patterns of DEGs in the Chinese fir root tips of CK vs. Al groups; Figure S6: Expression patterns of DEGs in the Chinese fir root tips of Al+MJ vs. Al+AIP groups; Table S1: Sequencing data statistics of *C. lanceolata* root tips; Table S2: Statistical table of comparison results for young *C. lanceolata* seedling roots; Table S3: The expression level of *C. lanceolata* root tips genes; Table S4: Protein database annotations of DEGs selected under different comparison groups; Table S5: Expression matrix of antioxidant enzymes, secondary metabolite synthesis, and cell wall modification DEGs under different comparison groups.
