# Supplementary material for: Phenolic Compounds Enhance Aluminum Tolerance in Chinese Fir (Cunninghamia lanceolata) by Regulating Reactive Oxygen Species Homeostasis and Cell Wall Properties Under Aluminum Stress
Source: Plants (Basel). 2025 Aug 26;14(17):2658. doi: 10.3390/plants14172658 (PMC12429914; doi:10.3390/plants14172658)
Supplement: Supplementary file 1 [file plants-14-02658-s001.zip › Supplementary figures.pdf]

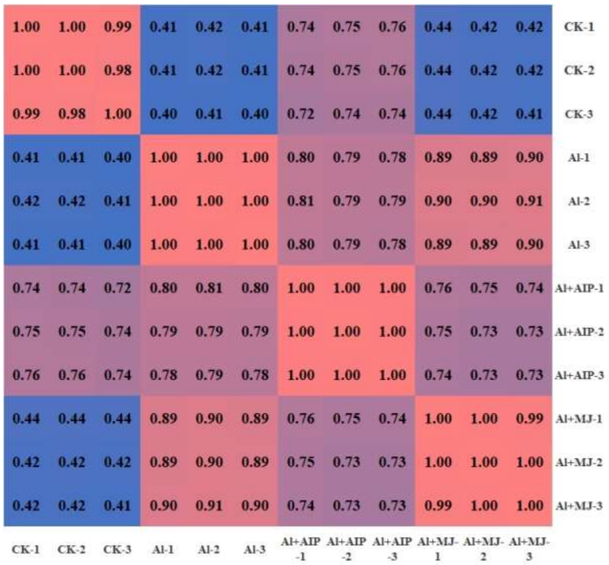

Figure S1. Heatmap of correlation between *C. lanceolata* root tips. The redder the color, the higher the correlation.

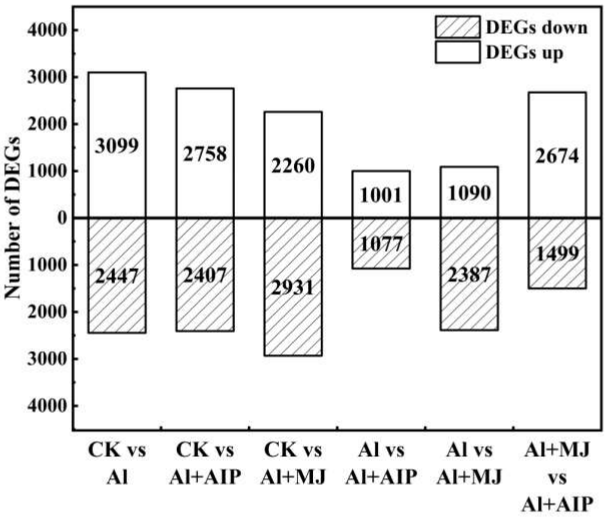

Figure S2. Number of upregulated and downregulated DEGs in different control groups of *C. lanceolata* seedling root tips.

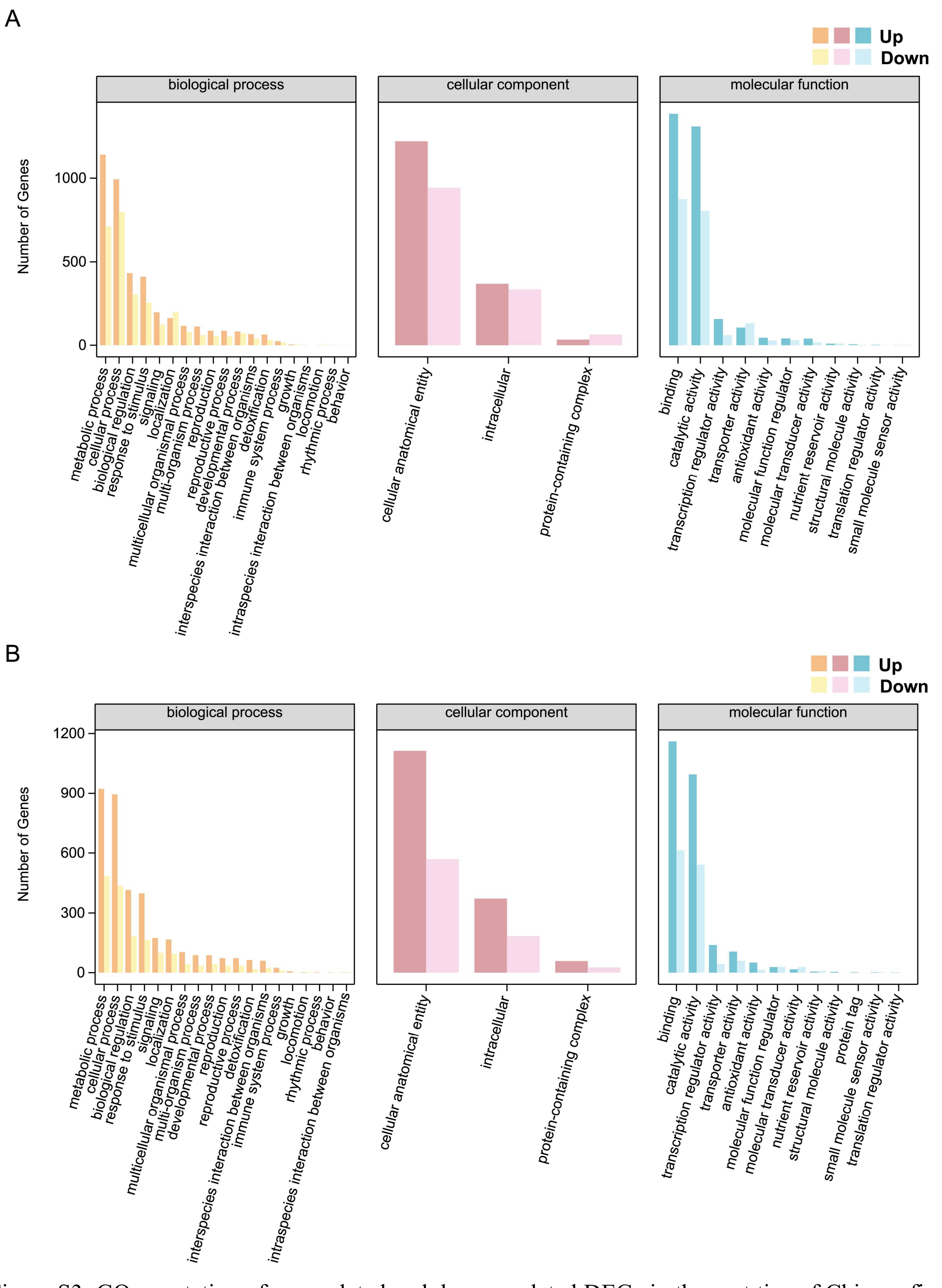

Figure S3. GO annotation of upregulated and downregulated DEGs in the root tips of Chinese fir. (A) GO enrichment analysis bar chart of CK vs. Al. (B) GO enrichment analysis bar chart of Al+MJ vs. Al+AIP. Yellow, pink, and blue represent biological process, cellular component, and molecular function, respectively. Darker columns represent the number of up-regulated DEGs, while lighter columns indicate the number of down-regulated DEGs.

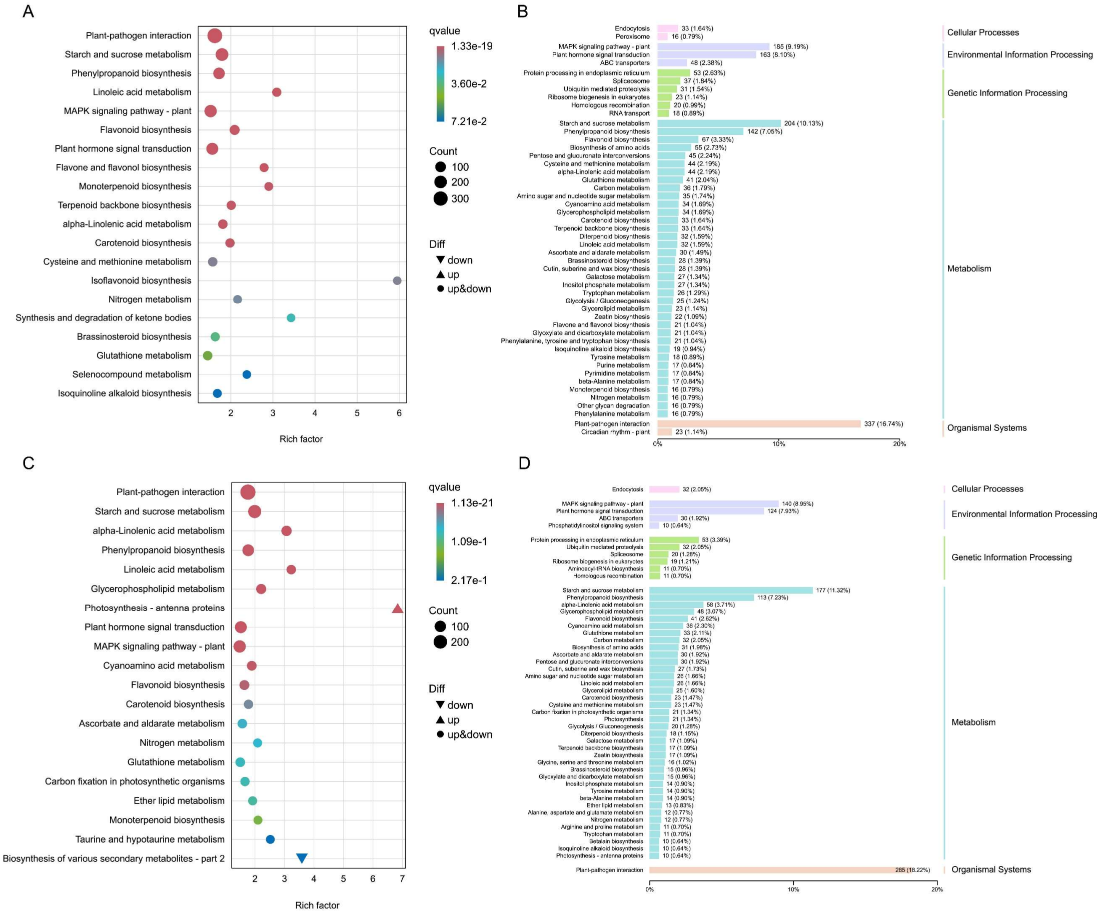

Figure S4. KEGG analysis of DEGs in Chinese fir root tips. KEGG pathway enrichment analysis of DEGs between CK vs. Al (A) and Al+MJ vs. Al+AIP (C). The x-axis and y-axis represent the enrichment factor and metabolic pathways, respectively. The size of the circles indicates the number of genes enriched in the pathway; larger circles indicate more genes. The smaller the qvalue, the redder the color, indicating that the enrichment significance of DEGs in that pathway is more reliable. Triangles represent upregulated DEGs, inverted triangles represent downregulated DEGs, and circles represent both up- and downregulated DEGs. KEGG classification analysis of DEGs between CK vs. Al (B) and Al+MJ vs. Al+AIP (D). Different colors represent different pathway types.

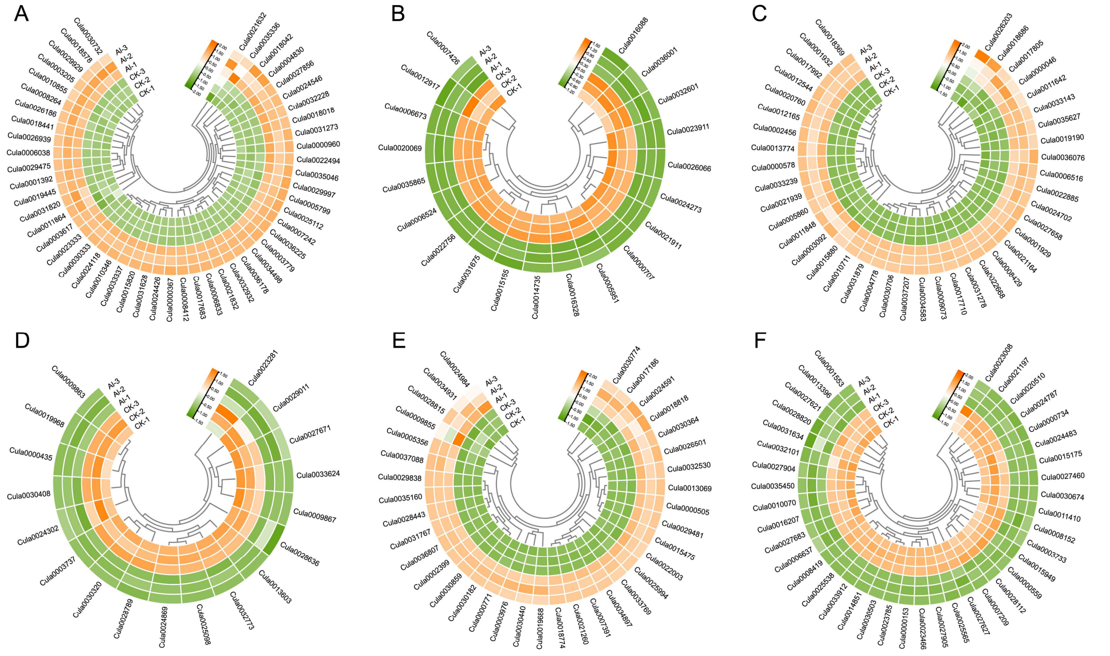

Figure S5. Expression patterns of DEGs in the Chinese fir root tips of CK vs. Al groups.(A)(B) Upregulated and downregulated expression levels of genes encoding antioxidant enzymes. (C)(D) Upregulated and downregulated expression levels of genes involved in secondary metab-olite synthesis. (E)(F) Upregulated and downregulated expression levels of genes involved in cell wall synthesis. All gene expression levels are expressed after normalization adjustment. Clustered by row, the orange gradient indicates upregulated expression levels, and the green gradient rep-resents downregulated expression levels.

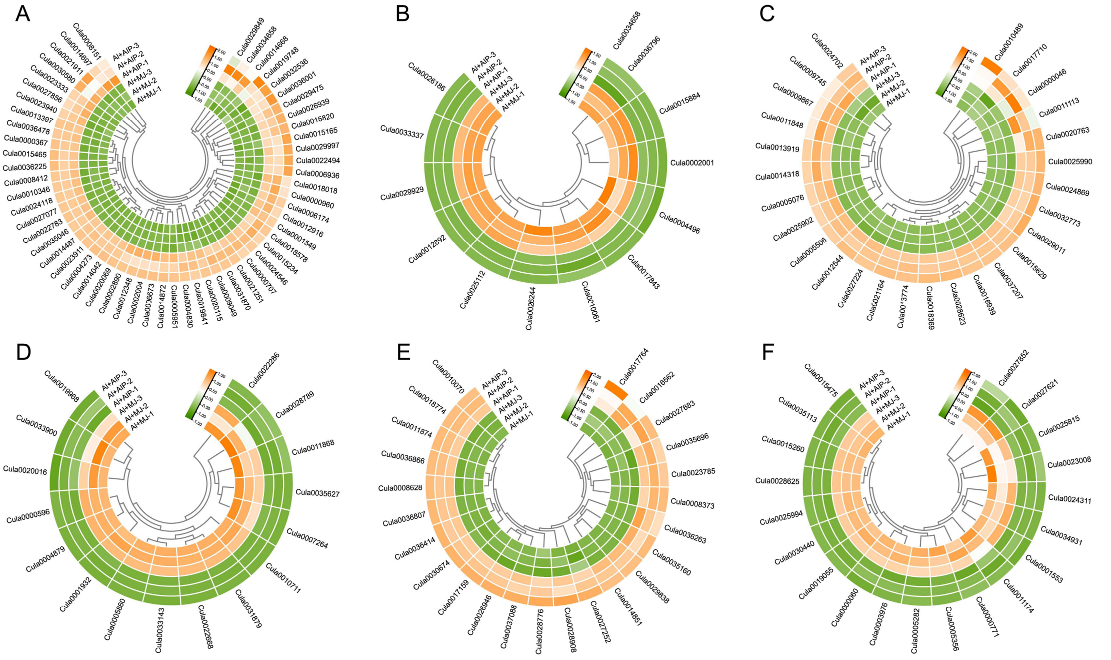

Figure S6. Expression patterns of DEGs in the Chinese fir root tips of Al+MJ vs. Al+AIP groups. (A)(B) Upregulated and downregulated expression levels of genes encoding antioxidant enzymes. (C)(D) Upregulated and downregulated expression levels of genes involved in secondary metab-olite synthesis. (E)(F) Upregulated and downregulated expression levels of genes involved in cell wall synthesis. All gene expression levels are expressed after normalization adjustment. Clustered by row, the orange gradient indicates upregulated expression levels, and the green gradient rep-resents downregulated expression levels.
